# BCL-XL Overexpression Protects Pancreatic β-Cells against Cytokine- and Palmitate-Induced Apoptosis

**DOI:** 10.3390/ijms24065657

**Published:** 2023-03-16

**Authors:** Atenea A. Perez-Serna, Reinaldo S. Dos Santos, Cristina Ripoll, Angel Nadal, Decio L. Eizirik, Laura Marroqui

**Affiliations:** 1 Instituto de Investigación, Desarrollo e Innovación en Biotecnología Sanitaria de Elche (IDiBE), Universidad Miguel Hernández de Elche, 03202 Elche, Alicante, Spain; 2 CIBER de Diabetes y Enfermedades Metabólicas Asociadas, Instituto de Salud Carlos III, Spain; 3 ULB Center for Diabetes Research, Medical Faculty, Université Libre de Bruxelles, 1070 Brussels, Belgium

**Keywords:** apoptosis, BCL-XL, Ca^2+^ signaling, cytokines, insulin secretion, palmitate, pancreatic β-cells

## Abstract

Diabetes is a chronic disease that affects glucose metabolism, either by autoimmune-driven β-cell loss or by the progressive loss of β-cell function, due to continued metabolic stresses. Although both α- and β-cells are exposed to the same stressors, such as proinflammatory cytokines and saturated free fatty acids (e.g., palmitate), only α-cells survive. We previously reported that the abundant expression of BCL-XL, an anti-apoptotic member of the BCL-2 family of proteins, is part of the α-cell defense mechanism against palmitate-induced cell death. Here, we investigated whether BCL-XL overexpression could protect β-cells against the apoptosis induced by proinflammatory and metabolic insults. For this purpose, BCL-XL was overexpressed in two β-cell lines—namely, rat insulinoma-derived INS-1E and human insulin-producing EndoC-βH1 cells—using adenoviral vectors. We observed that the BCL-XL overexpression in INS-1E cells was slightly reduced in intracellular Ca^2+^ responses and glucose-stimulated insulin secretion, whereas these effects were not observed in the human EndoC-βH1 cells. In INS-1E cells, BCL-XL overexpression partially decreased cytokine- and palmitate-induced β-cell apoptosis (around 40% protection). On the other hand, the overexpression of BCL-XL markedly protected EndoC-βH1 cells against the apoptosis triggered by these insults (>80% protection). Analysis of the expression of endoplasmic reticulum (ER) stress markers suggests that resistance to the cytokine and palmitate conferred by BCL-XL overexpression might be, at least in part, due to the alleviation of ER stress. Altogether, our data indicate that BCL-XL plays a dual role in β-cells, participating both in cellular processes related to β-cell physiology and in fostering survival against pro-apoptotic insults.

## 1. Introduction

Type 1 and type 2 diabetes are characterized by β-cell dysfunction and demise. In type 1 diabetes, β-cell apoptosis results from an autoimmune attack, where β-cells are exposed to proinflammatory cytokines that are released by infiltrating immune cells. Type 2 diabetes, on the other hand, is marked by metabolic-stress-mediated loss of functional β-cell mass, in which high levels of free fatty acids (e.g., palmitate) impair β-cell function and survival [1,2].

The BCL-2 family of proteins plays a critical role in pancreatic β-cell survival, with the balance between anti-apoptotic and pro-apoptotic BCL-2 proteins defining whether the cell will either follow the mitochondrial pathway of apoptosis or survive [3]. BCL-XL, a member of the BCL-2 anti-apoptotic proteins, can bind to and sequester BH3-only activators (i.e., Puma, Bim, and tBid), which will prevent BAX- and BAK-mediated mitochondrial apoptosis [4]. In addition, BCL-XL also prevents mitochondrial outer membrane permeabilization by retrotranslocating the pro-apoptotic protein BAX from the mitochondria into the cytosol [5]. Pro-apoptotic insults, such as proinflammatory cytokines and palmitate, can either directly or indirectly regulate β-cell BCL-XL expression. For instance, tumor necrosis factor-α down-regulates BCL-XL at both mRNA and protein levels [6], while IFNγ plus IL-1β [6,7], or palmitate [8], induce the expression of the BH3-only sensitizer DP5—which, in turn, binds to and represses BCL-XL, thereby favoring apoptosis.

BCL-XL promotes the survival of differentiating pancreatic progenitors from human-induced pluripotent stem cells [9]. Moreover, BCL-XL is key for the protection of β-cells against apoptotic stimuli, such as proinflammatory cytokines [6,10,11,12,13] and the endoplasmic reticulum (ER) stress inducer thapsigargin [11,14]. We have previously shown that pancreatic α-cells express much higher levels of BCL-XL than β-cells, which may contribute to α-cell resistance to palmitate. In fact, BCL-XL silencing sensitized α-cells to palmitate-induced apoptosis [15].

Due to its potential relevance to β-cell viability, two previous studies overexpressed BCL-XL with the aim of improving islet cell survival. The first showed that mouse islets overexpressing BCL-XL in β-cells were protected from thapsigargin-induced cell death [14]. Later, Holohan et al. showed that overexpression of BCL-XL in rat insulinoma cells (RIN-r) prevented the apoptosis induced by cytokines [10]. However, these prior studies were performed in rodents, and, to our knowledge, there have been no similar studies conducted on human models (e.g., human islets or β-cell lines). This is a crucial unmet need, as human islets/β-cells show major differences in their responses to stresses when compared to rodent islets/β-cells [16,17,18]. Against this background, our main objective in the present study was to achieve a step forward and to investigate whether BCL-XL overexpression could also protect human β-cells against the apoptosis induced by proinflammatory (i.e., IFNγ plus IL-1β) or metabolic (i.e., palmitate) insults. For this purpose, we overexpressed BCL-XL in two β-cell lines; namely, the rat insulinoma-derived INS-1E and the human EndoC-βH1 cells.

## 2. Results

### 2.1. BCL-XL Overexpression in Rat and Human β-Cell Models

**Figure 1:**
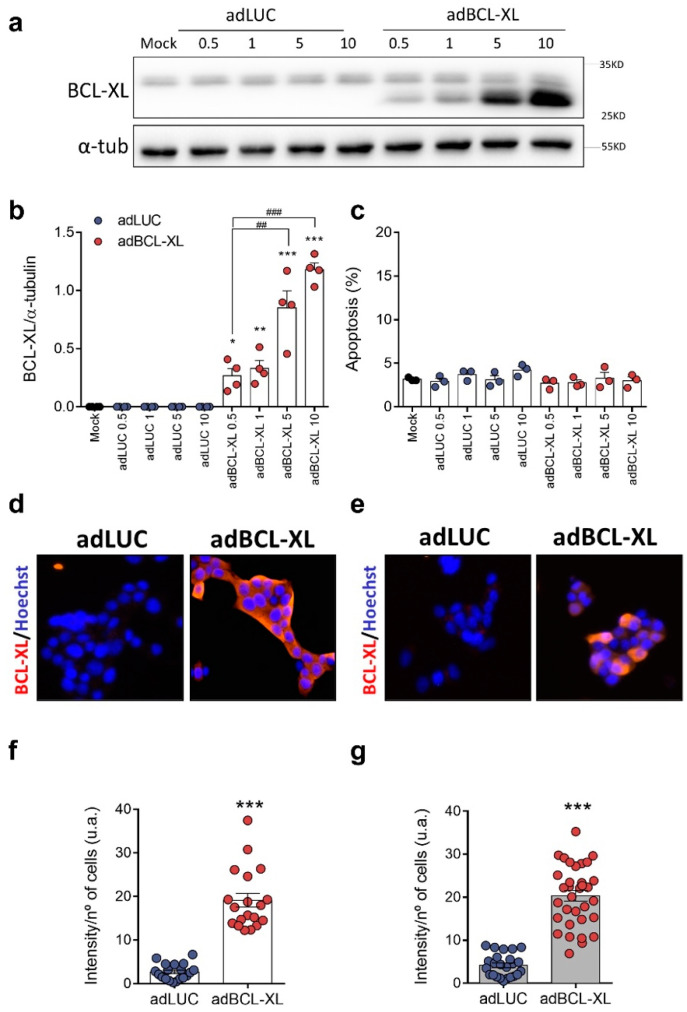
BCL-XL overexpression in rat and human β-cell lines. INS-1E (**a**–**d**,**f**) and EndoC-βH1 cells (**e**,**g**) were infected with adenoviral (ad) vectors encoding luciferase (adLUC, blue dots) or BCL-XL (adBCL-XL, red dots). After 48 h of recovery, protein expression and viability were assessed. (**a**,**b**) Protein expression was measured by Western blot. A representative image of four independent experiments is shown in (**a**), and densitometry results are presented for BCL-XL (**b**). (**c**) Apoptosis was assessed using HO/PI staining. Results are the means ± SEM of 3–4 independent experiments, where each dot represents an independent experiment. * *p* ≤ 0.05, ** *p* ≤ 0.01, and *** *p* ≤ 0.001 vs. Mock; ^##^ *p* ≤ 0.01 and ^###^ *p* ≤ 0.001 as indicated by bars. One-way ANOVA. (**d**–**g**) BCL-XL protein expression was analyzed by immunocytochemistry in INS-1E (**d**,**f**) and EndoC-βH1 cells (**e**,**g**). BCL-XL is shown in red and Hoechst 33342 in blue. Representative images (magnification ×20) are shown in (**d**,**e**) and densitometry results are presented for BCL-XL (**f**,**g**). Results are means ± SEM of 19–33 images, where each dot represents an image (4 independent experiments per cell line). *** *p* ≤ 0.001 vs. adLUC. Two-tailed unpaired Student’s *t*-test.

As it is known that adenoviral vectors can have a dose-dependent negative effect on rat islet cell survival, regardless of the gene that is overexpressed [19], we infected INS-1E cells with different multiplicities of infection (MOIs) and assessed cell viability 48 h from infection. Compared to adLUC-infected cells, BCL-XL expression was 4- (MOI 0.5) to 20-fold (MOI 10) higher in adBCL-XL-infected INS-1E cells (Figure 1a,b). No significant changes in apoptosis rate were observed upon infection with either adLUC or adBCL-XL (Figure 1c), suggesting that neither the adenoviral vectors nor the MOIs used induced cytotoxicity. Evaluation of BCL-XL expression by immunofluorescence showed that infection with MOI 5 adBCL-XL induced a 7- and 5-fold increase in BCL-XL expression in INS-1E and EndoC-βH1 cells, respectively (Figure 1d,e).

### 2.2. Modulation of Intracellular Ca^2+^ Signals and Glucose-Stimulated Insulin Secretion by BCL-XL

**Figure 2:**
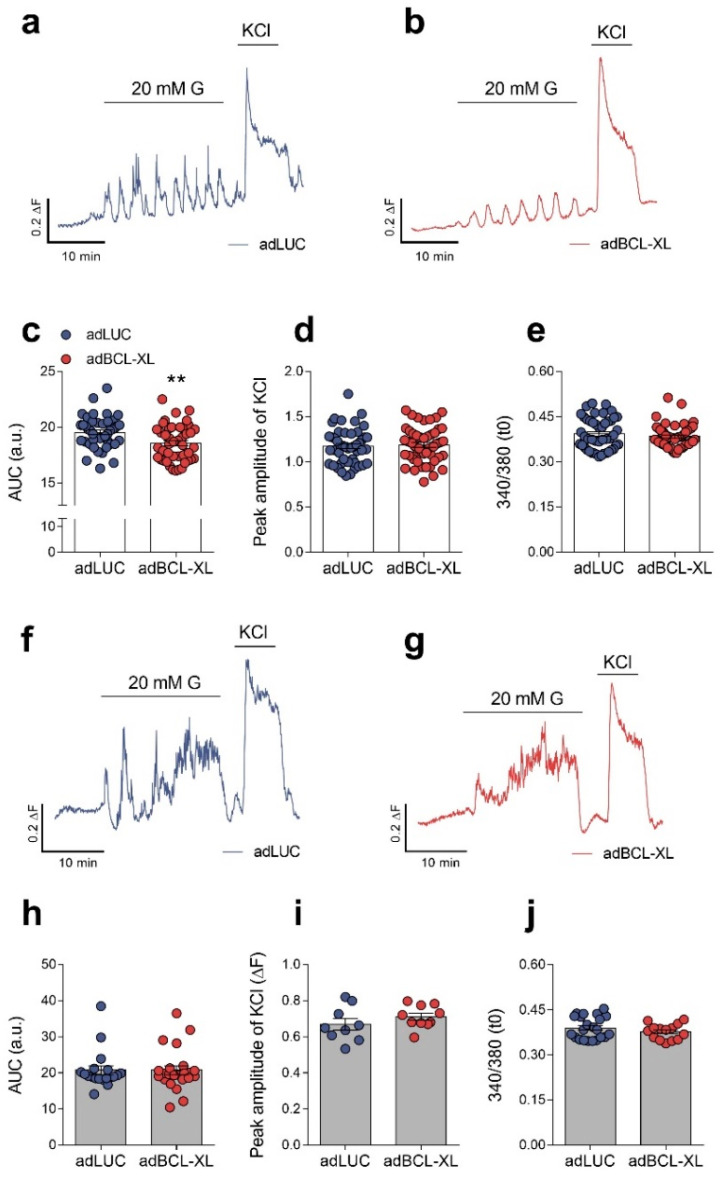
BCL-XL overexpression reduces intracellular Ca^2+^ oscillations in rat but not in human β-cells. INS-1E (**a**–**e**, white bars) and EndoC-βH1 cells (**f**–**j**, grey bars) were infected with adenoviral (ad) vectors encoding luciferase (adLUC, blue traces or dots) or rat BCL-XL (adBCL-XL, red traces or dots). After 48 h of recovery, intracellular Ca^2+^ dynamics was assessed using Fura-2AM fluorescence. Representative recordings of Fura-2AM Ca^2+^ fluorescence in INS-1E (**a**,**b**) and EndoC-βH1 cells (**f**,**g**). (**c**,**h**) Quantification of area under curve (AUC) in intracellular Ca^2+^ changes in response to 20 mM glucose (20 mM G) in INS-1E (**c**) and EndoC-βH1 cells (**h**). (**d**,**i**) Amplitude of the intracellular Ca^2+^ peak responses elicited by 20 mM KCl were measured in INS-1E (**d**) and EndoC-βH1 cells (**i**). (**e**,**j**) F340/F380 ratio under non-stimulatory glucose concentrations in INS-1E (**e**) and EndoC-βH1 cells (**j**). Results are the means ± SEM of 44–53 individual cell registers, where each dot represents a register (4–6 independent experiments per cell line). ** *p* ≤ 0.01 vs. adLUC. Two-tailed Student’s *t*-test.

BCL-XL has been shown to influence Ca^2+^ homeostasis in different cell types, including β-cells [14,20,21]. We tested whether BCL-XL overexpression affects intracellular Ca^2+^ oscillatory responses in rat and human β-cells by registering variations in intracellular Ca^2+^ concentration in response to a stimulatory glucose concentration (20 mM), followed by an extracellular depolarizing stimulus (30 mM KCl) (Figure 2a,b,f,g, Supplementary Figure S1). Non-stimulatory glucose concentrations were established at 3 mM for INS-1E cells and 0 mM for EndoC-βH1 cells. While BCL-XL overexpression induced a 5% reduction in Ca^2+^ oscillatory response to 20 mM glucose in INS-1E cells (Figure 2c), no changes were observed in human EndoC-βH1 cells overexpressing BCL-XL (Figure 2h). Other parameters related to intracellular Ca^2+^ concentration, such as Ca^2+^ peak amplitude upon KCl-induced depolarization (Figure 2d,i) and the ratio F340/F380 under non-stimulatory conditions (Figure 2e,j), were not altered by BCL-XL overexpression in both cell lines. These results indicate that the role of BCL-XL in regulating Ca^2+^ response to glucose in β-cells may be species-dependent, with a mild effect in rat but not human β-cells.

**Figure 3:**
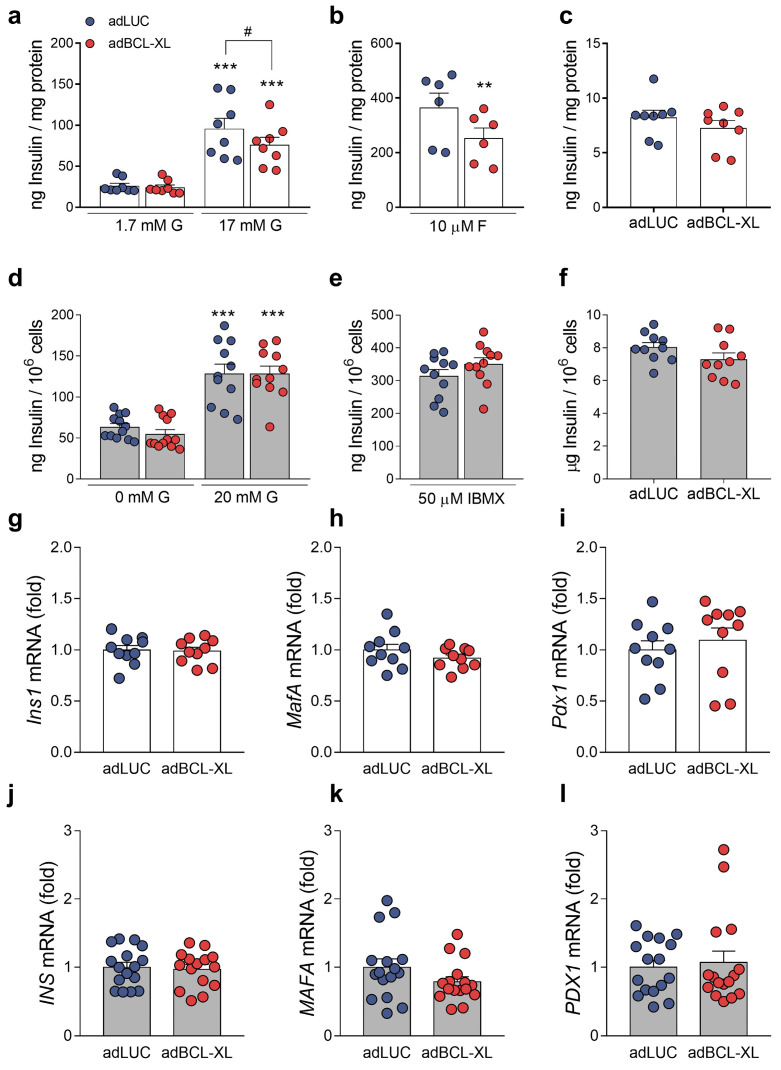
BCL-XL overexpression decreases insulin secretion in rat but not in human β-cells. INS-1E (**a**–**e**, white bars) and EndoC-βH1 cells (**f**–**j**, grey bars) were infected with adenoviral (ad) vectors encoding luciferase (adLUC, blue dots) or rat BCL-XL (adBCL-XL, red dots). After 48 h of recovery, glucose-stimulated insulin secretion was assessed. (**a**,**b**) Insulin secretion was measured at 1.7 and 17 mM glucose (**a**) or 17 mM + 10 μM Forskolin (10 μM F) (**b**). (**d**,**e**) Insulin secretion was measured at 0 and 20 mM glucose (**d**) or 20 mM + 50 μM IBMX (**e**). (**c**,**f**) Insulin content in cell-free lysates in INS-1E (**c**) and EndoC-βH1 cells (**f**). Insulin secretion and insulin content were measured by ELISA. (**g**–**i**) mRNA expressions of *Ins1* (**g**), *Mafa* (**h**), and *Pdx1* (**i**) were analyzed by RT-qPCR and normalized by *Gapdh* in INS-1E cells. (**j**–**l**) mRNA expressions of *INS* (**j**), *MAFA* (**k**), and *PDX1* (**l**) were analyzed by RT-qPCR and normalized by β-actin in EndoC-βH1 cells. Results are the means ± SEM of the 6 to 16 independent experiments, where each dot represents an independent experiment. (**a**,**d**) *** *p* ≤ 0.001 vs. low glucose (1.7 or 0 mM) and infected with the same adenoviral vector; ^#^ *p* ≤ 0.05 as indicated by bars. Two-way ANOVA. (**b**) ** *p* ≤ 0.01 vs. adLUC. Two-tailed Student’s *t*-test.

As changes in cytosolic Ca^2+^ concentrations play a key role in the control of insulin release from pancreatic β-cells [22,23], we determined whether BCL-XL overexpression changes β-cell function under basal conditions by assessing glucose-stimulated insulin secretion. In rat INS-1E cells, BCL-XL overexpression reduced insulin secretion in response to high glucose or in high glucose plus forskolin (an adenylate cyclase activator) by nearly 20% and 30%, respectively (Figure 3a,b). Insulin content remained unchanged (Figure 3c). Divergent from our data in INS-1E cells, insulin secretion in response to high glucose or high glucose plus IBMX (a phosphodiesterase inhibitor) was not modified by BCL-XL overexpression in EndoC-βH1 cells (Figure 3d,e). As in INS-1E cells, insulin content was not affected by BCL-XL overexpression (Figure 3f). These findings suggest that, in INS-1E cells overexpressing BCL-XL, the reduction in the Ca^2+^ oscillatory response under stimulatory conditions (i.e., 20 mM glucose) may result in the impairment of glucose-stimulated insulin secretion.

The mRNA expression of key genes for the maintenance of β-cell function and phenotype, namely *Ins1*/*INS*, *Mafa*/*MAFA*, and *Pdx1*/*PDX1*, did not change upon BCL-XL overexpression in INS-1E cells (Figure 3g–i) and EndoC-βH1 cells (Figure 3j–l). 

### 2.3. BCL-XL Overexpression Protects β-Cells against Cytokine- and Palmitate-Induced Apoptosis

**Figure 4:**
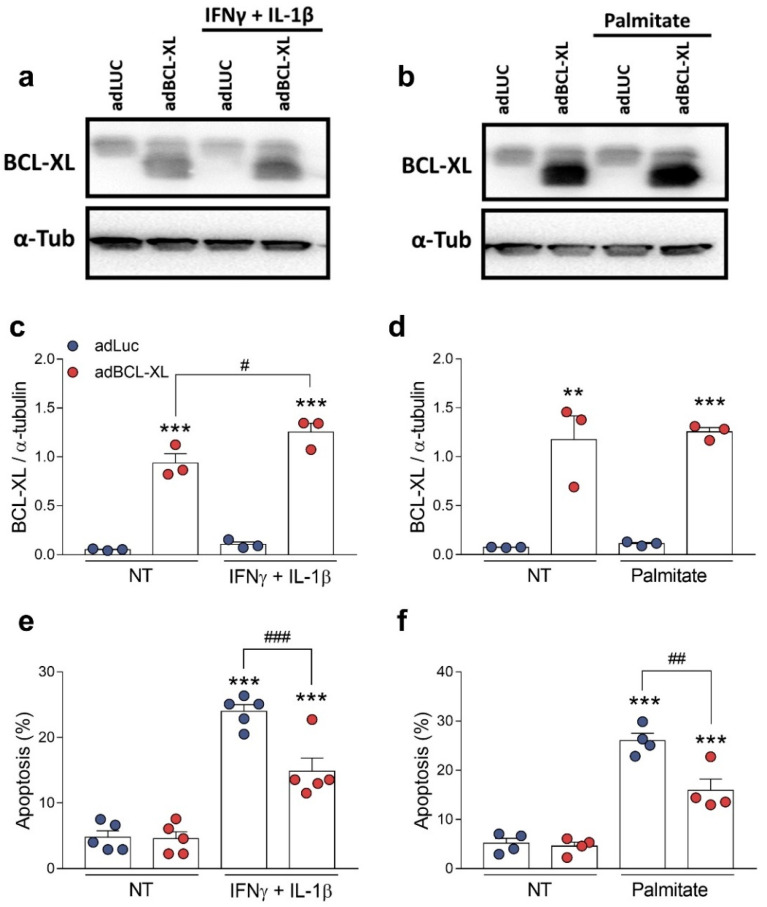
BCL-XL overexpression protects rat β-cells against cytokine- and palmitate-induced apoptosis. INS-1E cells were infected with adenoviral (ad) vectors encoding luciferase (adLUC, blue dots) or rat BCL-XL (adBCL-XL, red dots). After 48 h of recovery, cells were left untreated (NT) or treated with either IFNγ + IL-1β (10 and 100 U/mL, respectively) (**a**,**c**,**e**) or 0.5 mM palmitate (**b**,**d**,**f**) for 24 h. (**a**–**d**) Protein expression was measured by Western blot. Representative images of three independent experiments are shown in (**a**,**b**) and densitometry results are presented for BCL-XL (**c**,**d**). (**e**,**f**) Apoptosis was assessed using HO/PI staining. Results are the means ± SEM of the 3–5 independent experiments, where each dot represents an independent experiment. ** *p* ≤ 0.01 and *** *p* ≤ 0.001 vs. untreated (NT) and infected with the same adenoviral vector; ^#^ *p* ≤ 0.05, ^##^ *p* ≤ 0.01, and ^###^ *p* ≤ 0.001, as indicated by bars. Two-way ANOVA.

**Figure 5:**
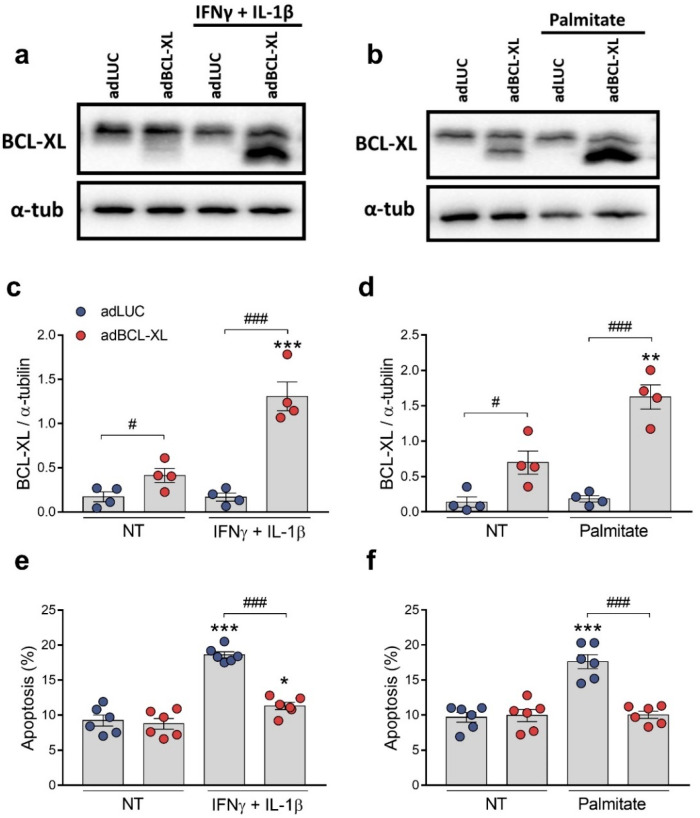
BCL-XL overexpression protects human β-cells from cytokine- and palmitate-induced apoptosis. EndoC-βH1 cells were infected with adenoviral (ad) vectors encoding luciferase (adLUC, blue dots) or rat BCL-XL (adBCL-XL, red dots). After 48 h of recovery, cells were left untreated (NT) or treated with either IFNγ + IL-1β (50 and 1000 U/mL, respectively) (**a**,**c**,**e**) or 0.5 mM palmitate (**b**,**d**,**f**) for 48 h. (**a**–**d**) Protein expression was measured by Western blot. Representative images of four independent experiments are shown in (**a**,**b**) and densitometry results are presented for BCL-XL (**c**,**d**). (**e**,**f**) Apoptosis was assessed using HO/PI staining. Results are the means ± SEM of the 4–6 independent experiments, where each dot represents an independent experiment. * *p* ≤ 0.05, ** *p* ≤ 0.01, and *** *p* ≤ 0.001 vs. untreated (NT) and infected with the same adenoviral vector; ^#^ *p* ≤ 0.05 and ^###^ *p* ≤ 0.001, as indicated by bars. Two-way ANOVA.

BCL-XL is known as an anti-apoptotic protein [4]. We next investigated whether BCL-XL overexpression would protect rodent and human β-cells against the apoptosis induced by proinflammatory (a mix of the cytokines IFNγ and IL-1β) or metabolic (palmitate) stimuli (Figure 4 and Figure 5). For this, we assessed cell viability using the DNA-binding dyes HO/PI upon treatment for 24 h (INS-1E cells) or 48 h (EndoC-βH1 cells). In rat INS-1E cells, β-cell death was induced by either cytokines (Figure 4a,c,e) or palmitate (Figure 4b,d,f). Furthermore, they were partially abrogated by BCL-XL overexpression, reaching about 40% protection in both conditions. 

Remarkably, human EndoC-βH1 cells overexpressing BCL-XL showed an apoptosis rate that was nearly 80% lower upon cytokine treatment than those found with the adLUC-infected cells (Figure 5a,c,e). Likewise, BCL-XL overexpression completely protected EndoC-βH1 cells from palmitate-induced apoptosis (Figure 5b,d,f). These data reinforce the role played by BCL-XL in human β-cell protection against different pro-apoptotic stimuli.

### 2.4. BCL-XL Overexpression Alleviates Cytokine- and Palmitate-Induced ER Stress in Human but Not in Rat β-Cells

**Figure 6:**
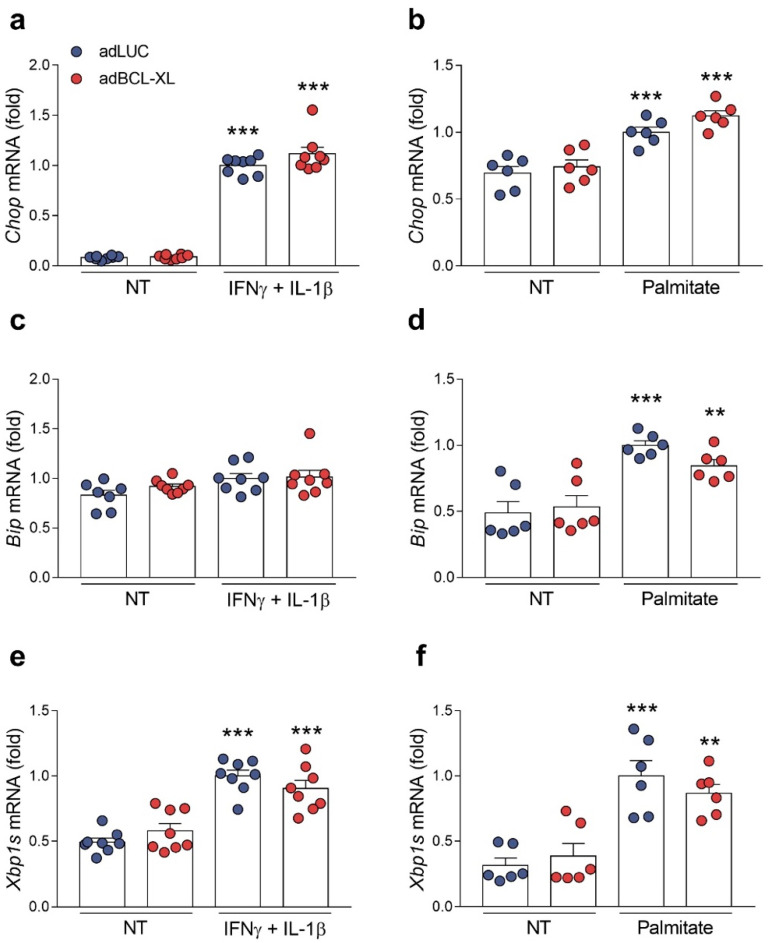
BCL-XL overexpression alleviates ER stress upon inflammatory or metabolic insults in rat β-cells. INS-1E cells were infected with adenoviral (ad) vectors encoding luciferase (adLUC, blue dots) or rat BCL-XL (adBCL-XL, red dots). After 48 h of recovery, cells were left untreated (NT) or treated with either IFNγ + IL-1β (10 and 100 U/mL, respectively) (**a**,**c**,**e**) or 0.5 mM palmitate (**b**,**d**,**f**) for 24 h. The mRNA expressions of *Chop* (**a**,**b**), *Bip* (**c**,**d**), and *Xbp1s* (**e**,**f**) were analyzed by RT-qPCR and normalized by *Gapdh*. Results are the means ± SEM of the 6 to 8 independent experiments. ** *p* ≤ 0.01 and *** *p* ≤ 0.001 vs. untreated (NT) and infected with the same adenoviral vector.

**Figure 7:**
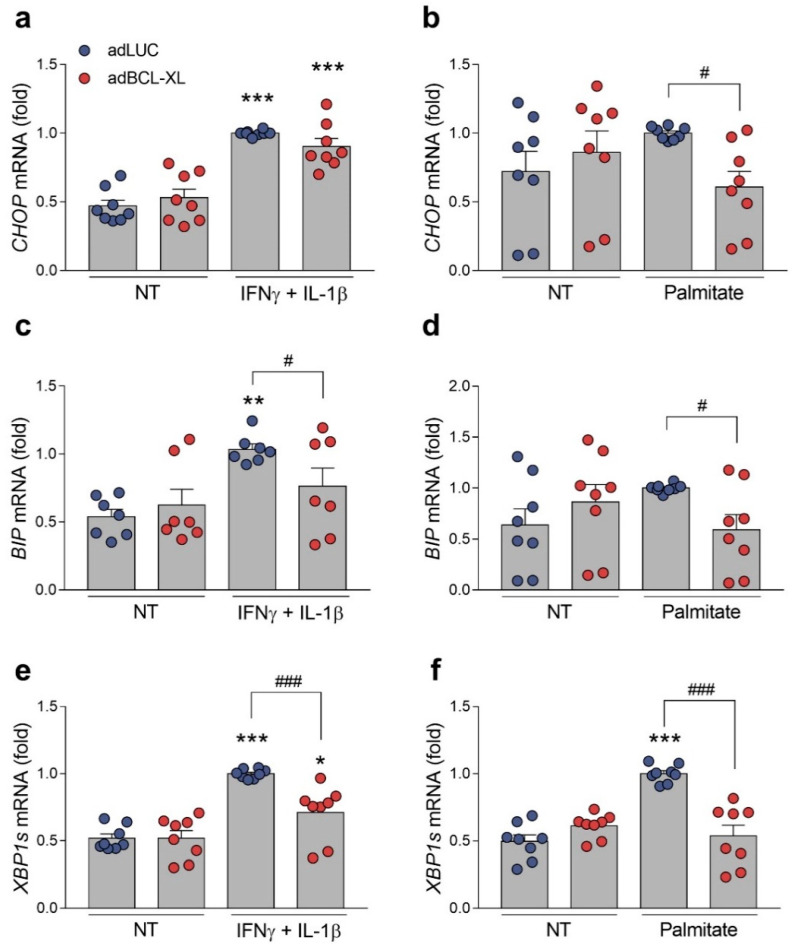
BCL-XL overexpression alleviates ER stress upon inflammatory or metabolic insults in human β-cells. EndoC-βH1 cells were infected with adenoviral (ad) vectors encoding luciferase (adLUC, blue dots) or rat BCL-XL (adBCL-XL, red dots). After 48 h of recovery, cells were left untreated (NT) or treated with either IFNγ + IL-1β (50 and 1000 U/mL, respectively) (**a**,**c**,**e**) or 0.5 mM palmitate (**b**,**d**,**f**) for 48 h. The mRNA expressions of *CHOP* (**a**,**b**), *BIP* (**c**,**d**), and *XBP1s* (**e**,**f**) were analyzed by RT-qPCR and normalized by β-actin. Results are the means ± SEM of the 7 to 8 independent experiments. * *p* ≤ 0.05, ** *p* ≤ 0.01, and *** *p* ≤ 0.001 vs. untreated (NT) and infected with the same adenoviral vector; ^#^ *p* ≤ 0.05 and ^###^ *p* ≤ 0.001, as indicated by bars. Two-way ANOVA.

As ER stress is a common mediator for β-cell apoptosis in both type 1 and type 2 diabetes [2], we assessed whether the protection achieved by BCL-XL overexpression is related to an alleviation of ER stress. For this purpose, we measured the expression of three key ER stress markers known to be induced in β-cells by cytokines [18] or palmitate [24]—namely, *Chop*/*CHOP*, *Bip*/*BIP*, and *Xbp1s*/*XBP1s*—in INS-1E (Figure 6) and EndoC-βH1 cells (Figure 7). Of note, these genes are, respectively, regulated by the three ER stress transducers PERK, ATF6, and IRE1α [25]. Overall, following proinflammatory (Figure 6a,c,e) and metabolic insults (Figure 6b,d,f), the stress-induced expression of *Chop*, *Bip*, and *Xbp1s* was found to be similar between the adLUC-infected and adBCL-XL-infected INS-1E cells. In EndoC-βH1 cells, however, BCL-XL overexpression led to a decrease in the expression of all of the ER stress markers cited above. In cytokine-treated cells, *BIP* and *XBP1s* levels decreased by 25–30%, while *CHOP* expression had a modest, non-significant 10% reduction (Figure 7a,c,e). A 40–50% reduction in the expression of all three ER stress genes was observed in BCL-XL-overexpressing cells when they were exposed to palmitate (Figure 7b,d,f). The overexpression of BCL-XL did not change *Chop*/*CHOP*’s, *Bip*/*BIP*’s, and *Xbp1s*/*XBP1s*’ mRNA expression in basal conditions (Figure 6 and Figure 7).

## 3. Discussion

In the present work, we show that BCL-XL overexpression protects both human and rodent β-cells against inflammatory- and metabolic stress-induced apoptosis. Depending on the species studied, the overexpression of BCL-XL differently affects intracellular Ca^2+^ oscillations and insulin secretion, as well as the expression of cytokine- and palmitate-induced ER stress markers in β-cells.

BCL-XL is an anti-apoptotic member of the BCL-2 family of proteins whose main function is to inhibit the mitochondrial outer membrane permeabilization by directly interacting with pro-apoptotic members of the BCL-2 family, including tBid and BAX [4]. However, the non-canonical roles of BCL-XL have been progressively unveiled in different cell types, indicating that this BCL-2 protein may also be involved in the control of other cellular processes, such as cell cycle and senescence [26,27], mitochondrial bioenergetics [28,29,30], and autophagy [31,32].

Beyond its role in the control of apoptosis, BCL-XL has been implicated in other aspects of β-cell physiology, such as Ca^2+^ homeostasis [14,21,33] and insulin secretion [14,21]. BCL-XL inhibition by two inhibitors, namely C6 and YC137, elicited Ca^2+^ fluctuations that resembled Ca^2+^ responses to glucose in human and mouse islet cells, as well as in the mouse MIN6 cell line. Similarly, islet cells from a β-cell-specific BCL-XL knockout mouse showed a significantly greater Ca^2+^ response induced by glucose [21]. While BCL-XL inhibition increased the intracellular Ca^2+^ signals in β-cells, BCL-XL overexpression reduced the intracellular Ca^2+^ responses to glucose in the islets from transgenic mice overexpressing very high levels (i.e., an over 10-fold increase) of human BCL-XL under the rat insulin promoter [14] and in rat INS-1E β-cells (present data). 

In the presence of substimulatory glucose concentrations, Luciani et al. reported that BCL2/BCL-XL antagonism acutely induced insulin secretion. Interestingly, the islets from the β-cell-specific BCL-XL knockout mice only displayed a modest, non-significant tendency toward increase in glucose-stimulated insulin release [21]. On the other hand, glucose-stimulated insulin secretion was reduced by 60% in islets from BCL-XL transgenic mice when compared with wild-type islets [14]. In addition, Pax4-induced BCL-XL expression in rat islets also resulted in the attenuation of insulin release in response to glucose [34]. Once again, our results in INS-1E cells concurred with the literature, as we observed that the overexpression of BCL-XL decreased the insulin secretion that was stimulated by glucose and by forskolin.

On this note, we did not detect changes in the intracellular Ca^2+^ oscillations or glucose-stimulated insulin secretion when BCL-XL was overexpressed in human β-cells. First, it seems unlikely that this lack of effect is due to the overexpression of the rat BCL-XL in human EndoC-βH1 cells. This is because, as discussed above, in the mouse islets overexpressing human BCL-XL, altered intracellular Ca^2+^ signals and insulin secretion were presented [14]. Alternatively, it is possible that a certain level of BCL-XL hyperexpression is necessary to induce alterations in Ca^2+^ homeostasis and β-cell secretory function. Zhou et al. observed that in mice in which BCL-XL expression was augmented, only 2- to 3-fold presented normal glucose tolerance and ex vivo glucose-stimulated insulin secretion. Conversely, animals in which BCL-XL expression surpassed a 10-fold increase displayed severe glucose intolerance, as well as impaired ex vivo insulin secretion and intracellular Ca^2+^ responses [14]. The fact that we observed a 2.5- to 5-fold induction in BCL-XL protein levels in EndoC-βH1 cells and a 15- to 20-fold in INS-1E cells supports the hypothesis of a threshold for BCL-XL overexpression.

BCL-XL plays a key role in β-cell survival and its silencing, either with small-interfering RNAs or with the pharmacological inhibition with small molecules that induced apoptosis under basal conditions in different β-cell models, such as in rat and mouse cell lines [6,8,12,13,21], primary rat and mouse islets/β-cells [12,21], and dispersed human islets [12,21]. BCL-XL expression increases during pancreatic specification from human pluripotent stem cells, which coincides with a decrease in cell death. Moreover, pancreatic progenitors, wherein BCL-XL was knocked down or chemically inhibited, showed increased cell death [9]. BCL-XL inhibition also sensitizes pancreatic cells to a wide range of deleterious stimuli, including thapsigargin [11], high concentrations of ribose [33], and proinflammatory cytokines [11,12]. In pancreatic α-cells, which are naturally protected from the apoptosis triggered by palmitate-induced, in part due to higher BCL-XL expression than in β-cells, BCL-XL knockdown did not induce apoptosis under basal conditions, but sensitized α-cells to palmitate. Interestingly, the rate of palmitate-induced apoptosis in BCL-XL-deficient α-cells was comparable to the rate observed in β-cells treated with palmitate [15]. In a mirror image of these findings, BCL-XL overexpression blunted stress-induced apoptosis in β-cells. First, Zhou et al. reported that the islets from transgenic mice overexpressing human BCL-XL were protected against thapsigargin-induced apoptosis [14]. Afterward, two other studies showed that BCL-XL prevents cell death upon exposure to a mix of proinflammatory cytokines [10,34]. In the present study, we not only confirm the anti-apoptotic role of BCL-XL against cytokine-triggered apoptosis, but also show, for the first time, that BCL-XL overexpression protects rat and human β-cells against the deleterious effects of palmitate. From a mechanistic perspective, this is an interesting finding. The pathogenesis of type 1 and type 2 diabetes is fundamentally different, where proinflammatory cytokines and palmitate trigger different biological processes and signaling pathways that will eventually impact β-cell dysfunction (immune-mediated vs. metabolic) and cell fate (massive vs. mild-to-moderate β-cell loss) in both forms of the disease [1,2]. However, despite inducing different pathways, cytokines and palmitate activate pro-apoptotic BH3-only proteins that repress BCL-XL, including DP5 and Bad [3]. In our β-cell models, it seems that an excess of BCL-XL overcomes the cytokine- or palmitate-induced activation of these BH3-only sensitizers, thereby leading to protection against apoptosis.

Due to its dual subcellular localization, both at the mitochondria and the ER, BCL-XL has been implicated in the protection from ER stress through different mechanisms, such as the sequestration of Bim, which prevents ER stress-induced Bim translocation to the ER [35,36]. This is in addition to the maintenance of ER membrane permeability to ER luminal proteins (e.g., BiP) [37]. In β-cells, previous studies have suggested that BCL-XL is a key player in controlling ER stress-induced apoptosis, as BCL-XL levels are critical to blunt thapsigargin-triggered apoptosis [11,14]. As prolonged ER stress contributes to both cytokine- and palmitate-induced β-cell apoptosis [25], we investigated whether BCL-XL-mediated protection against these stressors was associated with the alleviation of ER stress in rodent and human β-cells. Upon exposure of rat INS-1E cells to cytokines or palmitate, the increased mRNA expression of the ER stress markers *Bip*, *Chop*, and *Xbp1s* was not altered by BCL-XL overexpression. On the other hand, the cytokine- and palmitate-induced mRNA expression of *BIP*, *CHOP*, and *XBP1s* was mostly abrogated by BCL-XL overexpression in human EndoC-βH1 cells. Similar to our findings in human β-cells, mouse embryonic fibroblasts where BCL-XL was targeted to the ER presented decreased the expression of several ER stress markers, such XBP1s, CHOP, and ATF4, following thapsigargin insult—which may have conferred resistance to thapsigargin-induced cell death [38]. In this case, BCL-XL at the ER negatively regulated the inositol 1,4,5-trisphosphate receptor (IP_3_R)-mediated Ca^2+^ release from this organelle, which prevented ER Ca^2+^ depletion and ER stress [38]. Of note, previous studies reported that BCL-XL is mainly localized in the mitochondria, with minimal association with ER (i.e., a localization 6-fold higher in the mitochondria when compared to ER) in rodent β-cells [12,21]. Taken together, these findings suggest that the regulation of the ER stress response by BCL-XL may differ between rat and human β-cells. While it is possible that BCL-XL subcellular localization and its involvement in Ca^2+^ homeostasis might play a role in this regulation, further investigation will be needed to elucidate how BCL-XL modulates cytokine- and palmitate-induced ER stress in human but not in rat β-cells.

Although ER stress alleviation may account for the protection seen in BCL-XL-overexpressing human β-cells, it is likely that this protective effect also comes from the classical role played by BCL-XL as the gatekeepers of life and death. As the balance between anti- and pro-apoptotic BCL-2 proteins is key to define cell survival or death, BCL-XL overexpression may be just shifting the balance in favor of survival when cells are exposed to stressful conditions. This canonical, pro-survival role of BCL-XL would explain why rat β-cells overexpressing BCL-XL were partially protected from cytokine- and palmitate-induced apoptosis independently of the lack of effects that occurred following the ER stress response. Consistent with our data in β-cells, studies in other cell types have reported that BCL-XL overexpression protects from a myriad of stressors, such as viral infections [39], hydrogen peroxide [40], and lead exposure [41].

Based on our in vitro data, the next step would be to examine whether BCL-XL overexpression could prevent diabetes in animal models (e.g., NOD and *db*/*db* mice, respectively models for type 1 and type 2 diabetes). For this purpose, an interesting approach would be to use an adeno-associated virus(AAV)-based gene delivery system [42,43,44,45] to enhance BCL-XL expression specifically in β-cells of NOD and *db*/*db* mice, as well as to better evaluate the diabetes progression in these animals. In this case, gene therapy using AAV would require careful evaluation of the safety issues related to the expression of an anti-apoptotic protein in the long term. Among these safety issues, special attention should be given to increased susceptibility to viral infections and possible effects on tumorigenesis [39,46,47,48].

Collectively, our work adds to the evidence indicating that BCL-XL plays a dual role in β-cells, participating both in cellular processes related to β-cell physiology (e.g., Ca^2+^ homeostasis and insulin secretion) and survival. Moreover, it supports the notion that, depending on the amount of BCL-XL protein overexpressed, it is possible to protect β-cells against pro-apoptotic insults, without impairing β-cell function. This balanced scenario speaks in favor of the possibility of using BCL-XL as a therapeutic target for the treatment of diabetes. Importantly, we show for the first time that BCL-XL overexpression protects *human* β-cells without compromising insulin release, which sets the stage for the use of β-cell-specific BCL-XL overexpression as a potential therapeutic strategy in order to prevent β-cell loss during diabetes development.

## 4. Materials and Methods

### 4.1. Culture of EndoC-βH1 and INS-1E Cells

The human EndoC-βH1 β-cell line (research resource identifier (RRID):CVCL_L909, Univercell-Biosolutions, France) was grown attached to Matrigel/fibronectin-coated plates or glass coverslips as described before [49]. Cells were cultured in DMEM, containing 5.6 mM glucose, 10 mM nicotinamide, 5.5 μg/mL transferrin, 50 μM 2-mercaptoethanol, 6.7 ng/mL selenite, 2% BSA fatty acid free, 100 U/mL penicillin, and 100 μg/mL streptomycin.

The rat INS-1E β-cell line (RRID:CVCL_0351, provided by Dr. C. Wollheim, Department of Cell Physiology and Metabolism, University of Geneva, Geneva, Switzerland) was cultured in RPMI 1640 GlutaMAX-I, containing 10 mM HEPES, 1 mM sodium pyruvate, 50 μM 2-mercaptoethanol, 5% FBS, 100 U/mL penicillin, and 100 μg/mL streptomycin [50,51]. Both cell lines were kept at 37 °C in a humidified atmosphere of 5% CO_2_.

### 4.2. BCL-XL Overexpression

BCL-XL was overexpressed in INS-1E and EndoC-βH1 cells using a recombinant adenoviral vector containing the rat BCL2L1 gene (GeneBank: NM_001033672; SIRION Biotech, Gräfelfing, Germany). An adenovirus encoding Luciferase (adLUC; Ad-CMV-Luc2, SIRION Biotech, Gräfelfing, Germany) was used as a control. Infection with recombinant adenoviruses, adLUC or adBCL-XL, was performed as described elsewhere [52] and in different MOIs, i.e., the number of viruses that infect individual cells were used as indicated. Upon infection, cells were allowed to recover for 48 h before treatments.

### 4.3. Cell Treatments

Proinflammatory cytokine concentrations were used according to previously established dose–response experiments that were conducted in human and rodent cells [53,54,55]. The following concentrations were used: recombinant human IL-1β (R&D Systems, Cat. No. 201-LB/CF, Abingdon, UK) at 10 or 50 U/mL for INS-1E and EndoC-βH1 cells, respectively; recombinant human IFNγ (PeproTech, Cat. No. 300-02-250UG, Cranbury, NJ, USA) at 1000 U/mL for EndoC-βH1 cells; and recombinant rat IFNγ (R&D Systems, Cat. No. 585-IF) at 100 U/mL for INS-1E cells. For treatments in EndoC-βH1 cells, 2% FBS was added to the culture medium. 

Palmitate was prepared as described elsewhere [56]. Cells were treated with vehicle (ethanol) or 0.5 mM palmitate that were precomplexed to 0.67% BSA fatty acid free and 1% FBS. For EndoC-βH1 cells, treatment was performed in DMEM/Ham’s F12 (1:1, vol/vol) with supplements as described before [49,57].

### 4.4. Western Blot Analysis and Immunofluorescence

Cells were washed with cold PBS and lysed in Laemmli buffer. Immunoblot analysis was performed as described before [58] using the monoclonal rabbit anti-BCL-XL (1:1000) and the monoclonal mouse anti-α-tubulin (1:1000) antibodies. Densitometry analysis was performed with Image Lab software (version 4.1, Bio-Rad Laboratories, Madrid, Spain). 

Immunofluorescence was performed as previously described [59,60]. Cells were incubated overnight with a monoclonal rabbit anti-BCL-XL antibody (1:200). Alexa Fluor 568 polyclonal goat anti-mouse IgG (1:500) was applied for 1 h. Upon nuclei staining with Hoechst 33342, coverslips were mounted with a fluorescent mounting medium (DAKO) and the immunofluorescence was observed with an inverted fluorescence microscope (Nikon Eclipse TE-2000-V) equipped with a digital camera (Nikon DMX 1200C) and a Cool led PE-300 wheel as the fluorophore excitor. Images were acquired at ×20 magnification and analyzed using the open-source FIJI software (version 2.0).

See Supplementary Table S1 for more information about the antibodies used therein.

### 4.5. mRNA Extraction and Real-Time PCR

Poly(A)+ mRNA extraction was performed using Dynabeads mRNA DIRECT kit (Invitrogen, Madrid, Spain), following the manufacturer’s instructions. The High-Capacity cDNA Reverse Transcription Kit (Applied Biosystems, Foster City, CA, USA) was used to synthesize cDNA. Quantitative PCR was performed using the CFX96 Real Time System (Bio-Rad Laboratories, Madrid, Spain) [61]. The CFX Manager Version 1.6 (Bio-Rad Laboratories, Madrid, Spain) was used to analyze the values, which were expressed as relative expression in respect of control values (2^−ΔΔCt^) [62] using *Gapdh* and β-actin as the housekeeping genes for rat and human samples, respectively. Normalization was performed as follows: expression values were corrected by the housekeeping genes (i.e., *Gapdh* or β-actin, depending on the species). Afterwards, these values were normalized by the means of values obtained in control samples (considered as 1). In Figure 3, we normalized via the adLUC-infected cells, while in Figure 6 and Figure 7, values were normalized by the adLUC-infected cells treated with either cytokines or palmitate.

The primers used herein are listed in the Supplementary Table S2.

### 4.6. Intracellular Ca^2+^ Analysis

For intracellular Ca^2+^ measurements, cells were loaded with 2 ng/mL Fura-2AM (Invitrogen) for 1 h at room temperature in a humidified atmosphere. Fluorescence recordings were performed as described before [61]. Coverslips were perfused at a constant rate with a Krebs-Ringer solution containing 141 mM NaCl, 5.5 mM KCl, 1 mM MgCl_2_, 2 mM CaCl_2_, 20 mM HEPES, and pH 7.4. Basal fluorescence was determined in the absence of stimulus (3 mM glucose for INS-1E cells and 0 mM glucose for EndoC-βH1 cells) for 5 min before perfusing a solution with a stimulatory glucose concentration (20 mM glucose for both INS-1E and EndoC-βH1 cells). Fluorescence was determined for 15 min after each stimulus. Glucose-independent cell depolarization was elicited with high extracellular 30 mM KCl solution for 5 min, which was used as a positive control. Intracellular Ca^2+^ oscillations were recorded using an inverted fluorescence microscope (Zeiss Axiovert 200, Jana, Germany) equipped with a polychromator (TILL Photonics) to ensure 340- and 380-nm wavelength emission. Data were acquired with a Hamamatsu EMC9100 digital camera every 2.5 s and plotted with Aquacosmos version 2.6 (Hamamatsu Photonics, Massy, Francia). Ca^2+^ entry was assessed as an increase in the ratio of fluorescence at 340 and 380 nm (F340/F380) and analyzed as previously described [63] using GraphPad Prism version 7.0 (GraphPad Software, La Jolla, CA, USA). Intracellular Cas^2+^ variations were analyzed as changes in the area under the curve (AUC).

### 4.7. Glucose-Stimulated Insulin Secretion

Glucose-stimulated insulin secretion in INS-1E and EndoC-βH1 cells was performed as previously described [51,64]. Briefly: before glucose stimulation, INS-1E cells were preincubated for 1 h in glucose-free RPMI 1640 GlutaMAX-I medium and then for 30 min in a Krebs-Ringer solution (as described in [50]). For the EndoC-βH1 cell line, cells were preincubated for 1 h in Krebs-Ringer buffer (115 mM NaCl, 5 mM KCl, 1 mM MgCl_2_, 1 mM CaCl_2_, 24 mM NaHCO_3_, 10 mM HEPES, pH 7.4, and 0.1% BSA). At the end of this incubation, cells were sequentially stimulated with low glucose (1.7 or 0 mM), high glucose (17 or 20 mM) and then high glucose + forskolin (INS-1E cells) or high glucose + IBMX (EndoC-βH1 cells) for 30 min (INS-1E cells) or 1 h (EndoC-βH1 cells) (each stimulation). After each stimulatory period, the incubation medium was collected, placed onto ice and centrifuged at 700× *g*, 5 min at 4 °C. The supernatant was transferred into a fresh tube and stored at −80 °C until required for insulin measurements. For insulin content, cells were washed in cold PBS and then lysed in either an acid-ethanol solution (95% ethanol + 5% HCl) (INS-1E cells) or cell lysis solution (137 mM NaCl, 1% Glycerol, 0.1% Triton X100, 2 mM EGTA, 20 mM Tris pH 8.0 and protease inhibitor cocktail) (EndoC-βH1 cells). Insulin secretion and content were measured using a rat or a human insulin ELISA kit (Mercodia, Uppsala, Sweden), following the manufacturer’s instructions. Forskolin and IBMX (Sigma-Aldrich, Barcelona, Spain) were prepared by dissolution in DMSO and used at concentrations of 10 μM and 50 μM, respectively.

### 4.8. Assessment of Cell Viability

Cell viability was assessed by fluorescence microscopy using the DNA-binding dyes Hoechst 33342 (HO) and propidium iodide (PI), as previously described [50,65]. A minimum of 600 cells was counted for each experimental condition. Viability was evaluated by two independent observers, one of whom was unaware of sample identity. Agreement in results between observers was >90%. This method has been extensively validated and correlates closely to other methods to measure apoptosis, including electron microscopy, cytochrome *c* release, cleaved caspases 3 and 9, BAX activation, as well as the determinations of histone-complexed DNA fragments by ELISA, MTT assay, and caspase 3/7 activity [7,12,24,64,66,67,68,69,70].

### 4.9. Data Analysis

The GraphPad Prism 7.0 software (GraphPad Software, La Jolla, CA, USA) was used for statistical analyses. The Shapiro–Wilk normality test was used to determine the normal distribution. Except as otherwise indicated in the figure legend, results are shown as the mean ± SEM of the independent experiments (i.e., considering INS-1E or EndoC-βH1 cells from different passages as *n * =  1). Statistical analyses were performed using Student’s *t*-test or two-way ANOVA, as stated in the figure legends. One- and two-way ANOVA were followed by Dunnett’s test as the post hoc analysis. The *p* values ≤ 0.05 were considered statistically significant.

## Data Availability

Not applicable.

## Supplementary Materials

The following supporting information can be downloaded at: https://www.mdpi.com/article/10.3390/ijms24065657/s1.
