# Supplementary material for: BCL-XL Overexpression Protects Pancreatic β-Cells against Cytokine- and Palmitate-Induced Apoptosis
Source: Int J Mol Sci. 2023 Mar 16;24(6):5657. doi: 10.3390/ijms24065657 (PMC10056015; doi:10.3390/ijms24065657)
Supplement: Supplementary file 1 [file ijms-24-05657-s001.zip › ijms-2207145-supplementary.pdf]

Supplementary Material

BCL-XL overexpression protects pancreatic β-cells against cytokine- and palmitate-induced apoptosis

Atenea A. Perez-Serna, Reinaldo S. Dos Santos, Cristina Ripoll, Angel Nadal, Decio L. Eizirik, Laura Marroqui

Supplementary Table S1. List of antibodies used in this study.

Target antigen	Antibody name	Manufacturer and Catalogue number (Cat. no)	Species raised in	Dilution	RRID
BCL-XL	Bcl-xL (54H6) Rabbit mAb	Cell Signaling; Cat no. 2764	Rabbit, monoclonal	1:1000 (WB)<br>1:2000 (ICC)	AB_2228008
α-tubulin	Monoclonal Anti α-Tubulin antibody	Sigma; Cat no. T9026	Mouse, monoclonal	1:5000 (WB)	AB_477593
Goat anti-mouse IgG	Goat Anti-Mouse IgG (H+L) HRP Conjugate antibody	Bio-rad; Cat no. 170-6516 Goat	Goat, polyclonal	1:5000 (WB)	AB_11125547
Goat anti-rabbit IgG	Goat Anti-Rabbit IgG (H+L) HRP Conjugate antibody	Bio-rad; Cat no. 170-6515 Goat	Goat, polyclonal	1:5000 (WB)	AB_11125142
Alexa Fluor 568 goat anti-mouse IgG	Goat anti-Mouse IgG (H+L) Highly Cross-Adsorbed Secondary Antibody	Invitrogen; Cat no. A-11031	Goat, polyclonal	1:5000 (ICC)	AB_144696

Supplementary Table S2. List of primers used in this study.

	Forward	Reverse
	**Sequence (5'-3')**	**Sequence (5'-3')**
β-actin	CTGTACGCCAACACAGTGCT	GCTCAGGAGGAGCAATGATC
INS	GCTTCTTCTACACACCCAAGAC	CCACAATGCCACGCTTCT
PDX1	AAAGCTCACGCGTGGAAA	GCCGTGAGATGTACTTGTTGA
MAFA	TACAGGACGTGGACACCA	GTTCTCCGCTCAACCTCAG
BIP	ACCTCAGTCTCCCAGCTAAT	CCCTTGCCTGAGTAAAGATGT
CHOP	AACGGAAACAGAGTGGTCATT	GCTTGAGCCGTTCATTCTCT
XBP1s	CCGCAGCAGGTGCAGG	GAGTCAATACCGCCAGAATCCA

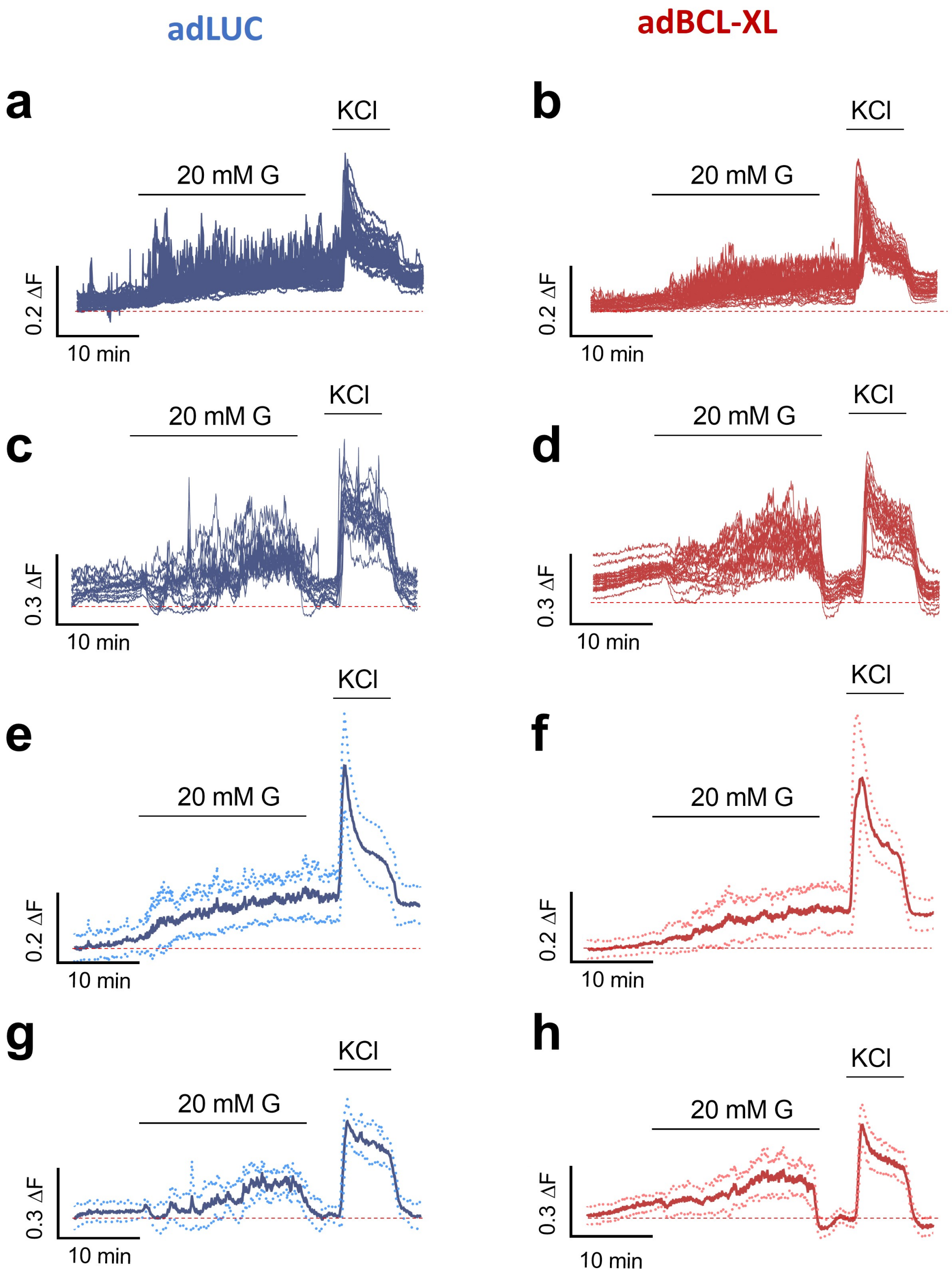

Supplementary Figure S1. BCL-XL overexpression reduces intracellular Ca^{2+} oscillations in rat but not in human β-cells. INS-1E (**a**,**b**,**e**,**f**) and EndoC-βH1 cells (**c**,**d**,**g**,**h**) were infected with adenoviral (ad) vectors encoding luciferase (adLUC, blue traces) or rat BCL-XL (adBCL-XL, red traces). After 48 h of recovery, intracellular Ca^{2+} dynamics was assessed using Fura-2AM fluorescence. (**a**–**d**) Recordings of Fura-2AM Ca^{2+} fluorescence in 44-53 individual cells, where each line represents a register (4-6 independent experiments per cell line). (**e**–**h**) Results are means ± SD (dotted lines) from data presented in (**a**–**d**). The red dotted lines in the bottom indicate the baseline.
